# Supplementary material for: Combined Biological and Numerical Modeling Approach for Better Understanding of the Cancer Viability and Apoptosis
Source: Pharmaceutics. 2023 May 31;15(6):1628. doi: 10.3390/pharmaceutics15061628 (PMC10302770; doi:10.3390/pharmaceutics15061628)
Supplement: Supplementary file 1 [file pharmaceutics-15-01628-s001.zip › pharmaceutics-2341049-supplementary.pdf]

Supplementary Data

Combined Biological and Numerical Modeling Approach for Better Understanding of the Cancer Viability and Apoptosis

Marko Živanović[1, *], Marina Gazdić Janković[2], Amra Ramović Hamzagić[2], Katarina Virijević[1], Nevena Milivojević[1], Katarina Pecić[1], Dragana Šeklić[1], Milena Jovanović[3], Nikolina Kastratović[2], Ana Mirić[1], Tijana Đukić[1], Ivica Petrović[2], Vladimir Jurišić[2], Biljana Ljujić[2] , Nenad Filipović[4, 5]

[1] Institute for Information Technologies Kragujevac, University of Kragujevac, Jovana Cvijića bb, 34000 Kragujevac, Serbia

[2] Faculty of Medical Sciences, University of Kragujevac, Svetozara Markovića 69, 34000 Kragujevac, Serbia

[3] Faculty of Sciences, University of Kragujevac, Radoja Domanovića 12, 34000 Kragujevac, Serbia

[4] Faculty of Engineering, University of Kragujevac, Sestre Janjić 6, 34000 Kragujevac, Serbia

[5] Bioengineering Research and Development Center (BioIRC), Prvoslava Stojanovica 6, 34000 Kragujevac, Serbia

* Corresponding author: Marko Živanović, Institute for Information Technologies Kragujevac, University of Kragujevac, Kragujevac, Serbia
zivanovicmkg@gmail.com

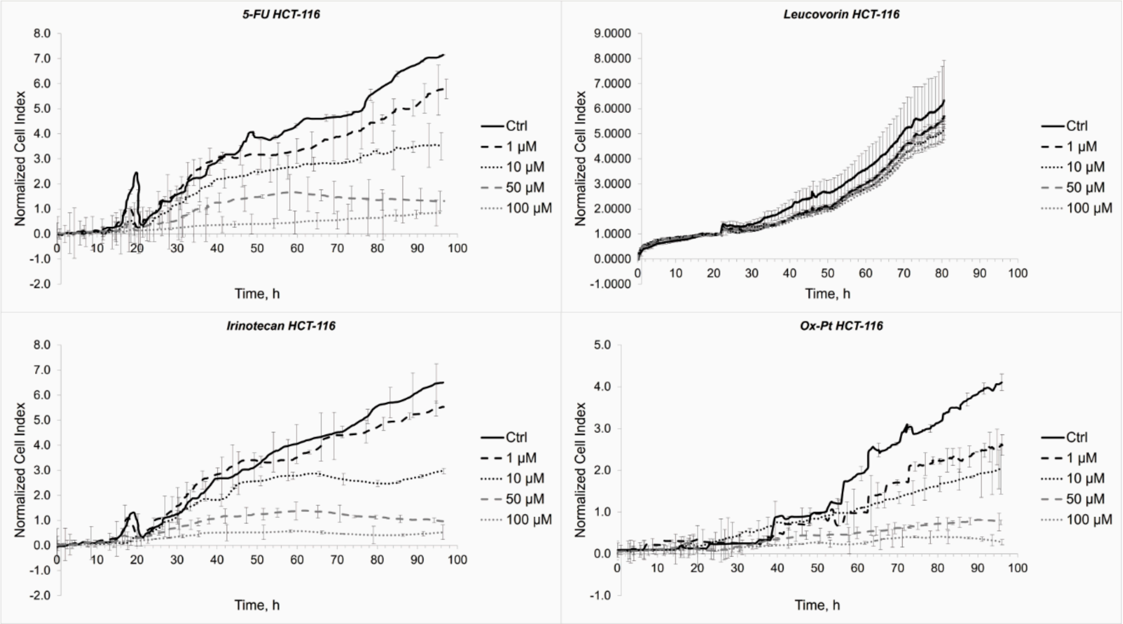

Figure S1. Normalized cell index (NCI) kinetics of the HCT-116 exposed to chemotherapeutics compared to control (untreated cells). The dose- and time-depended decrease of NCI, and thus of cell viability.

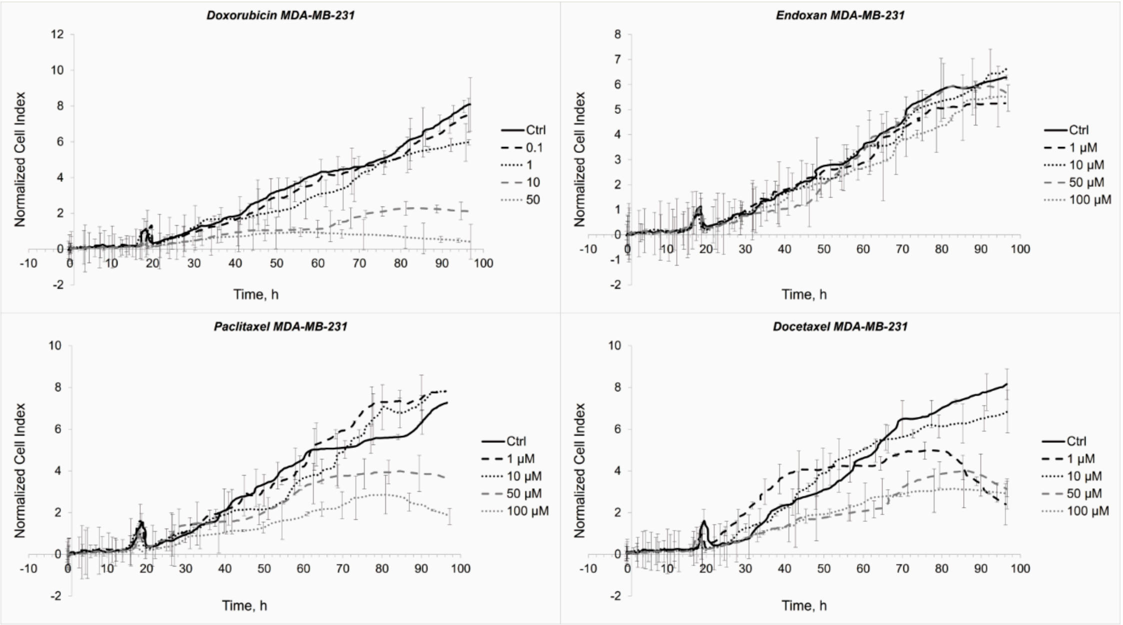

Figure S2. Normalized cell index (NCI) kinetics of the MDA-MB-231 exposed to chemotherapeutics compared to control (untreated cells). The dose- and time-depended decrease of NCI, and thus of cell viability.

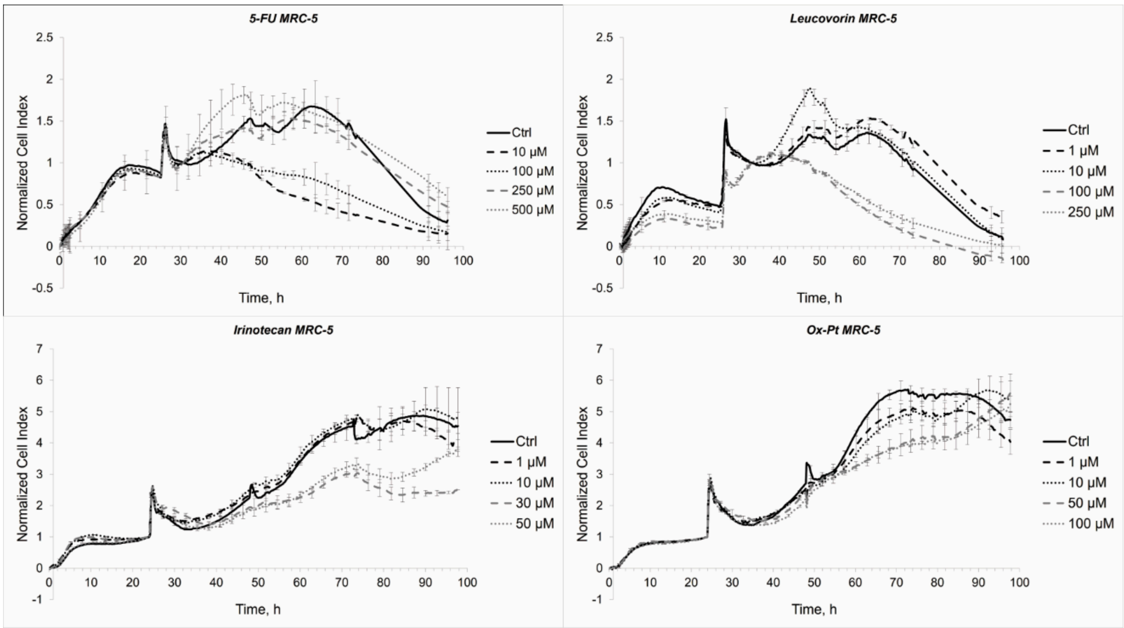

Figure S3. Normalized cell index (NCI) kinetics of the MRC-5 exposed to chemotherapeutics used for colon cancer treatment compared to control (untreated cells). The dose- and time-depended decrease of NCI, and thus of cell viability.

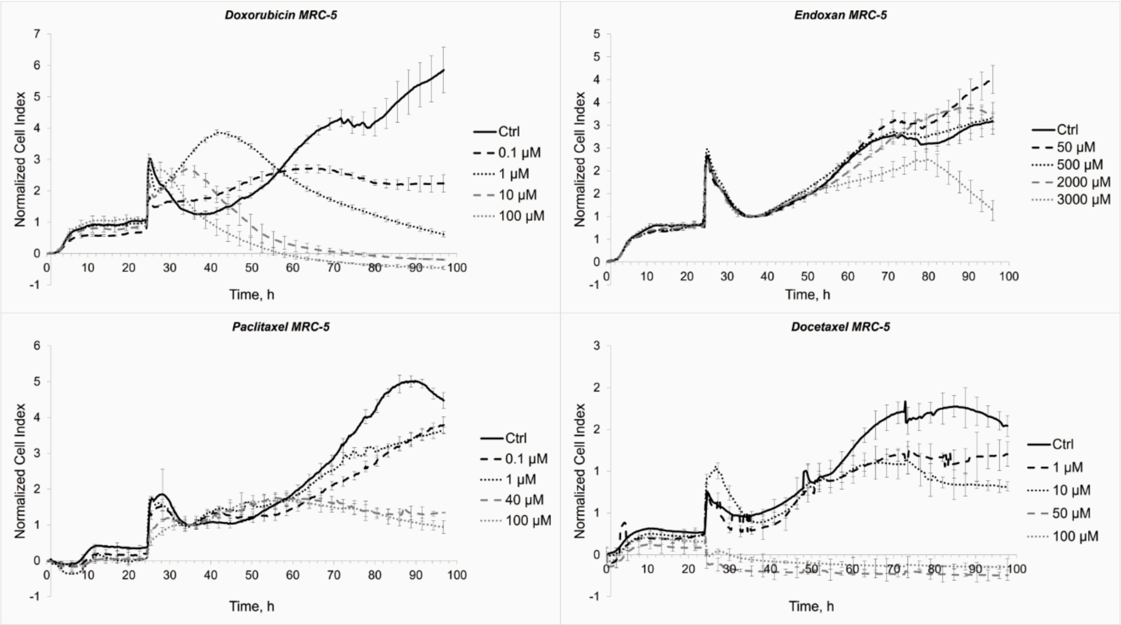

Figure S4. Normalized cell index (NCI) kinetics of the MRC-5 exposed to chemotherapeutics used for breast cancer treatment compared to control (untreated cells). The dose- and time-depended decrease of NCI, and thus of cell viability.

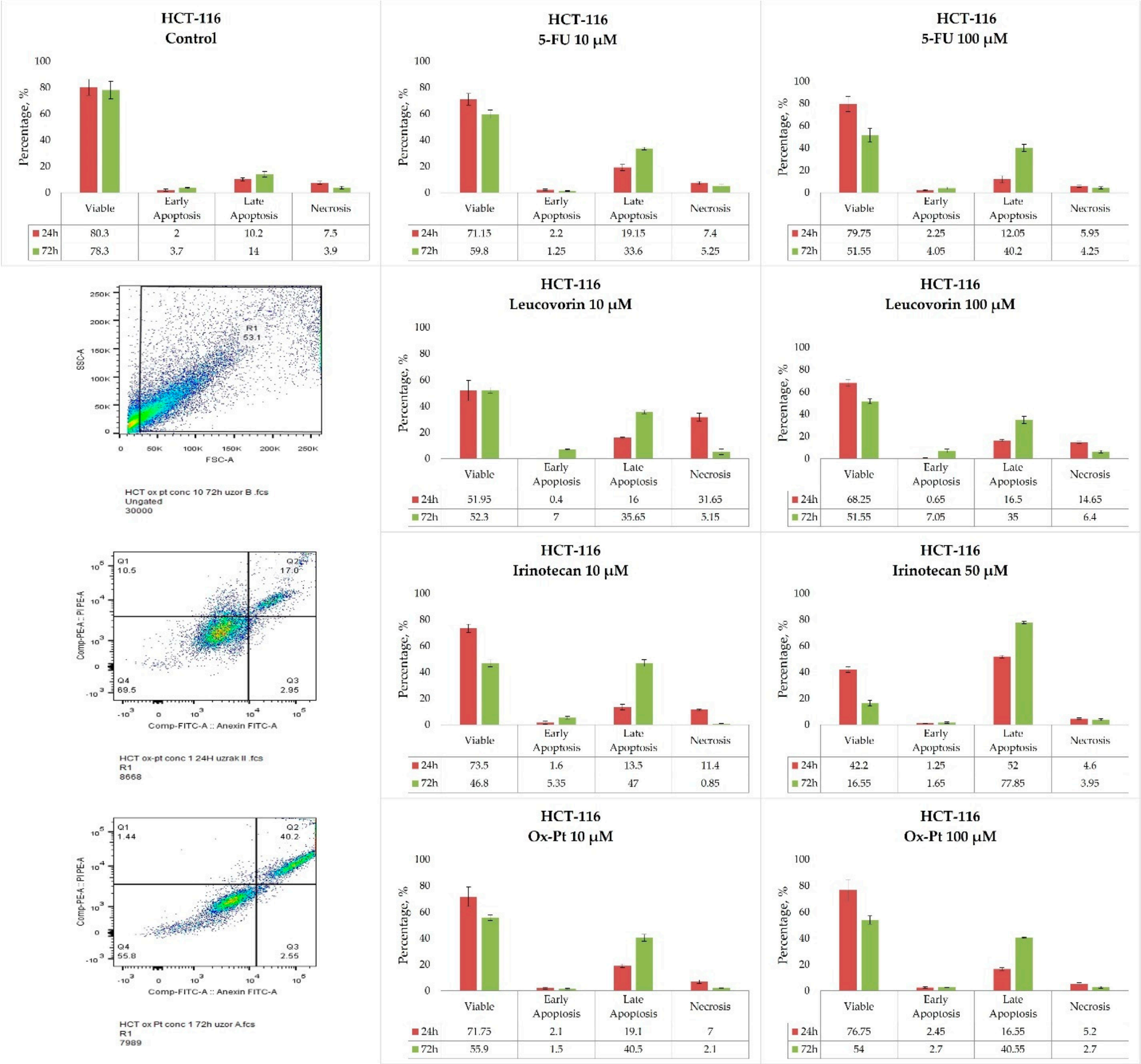

Figure S5. The effects of chemotherapeutics on apoptosis rate in HCT-116 cells. Inlet – Representative dot plots illustrate population of viable (ANNV- PI-), early apoptotic (Ann V+ PI-), late apoptotic (AnnV+ PI+), and necrotic (AnnV- PI+) cells treated with Ox-Pt. Apoptosis of untreated as well as chemotherapeutic-treated cells, analyzed by flow cytometry using Annexin V (FITC) and propidium iodide double staining. The data are presented as means ± SEM of a three-independent experiments.

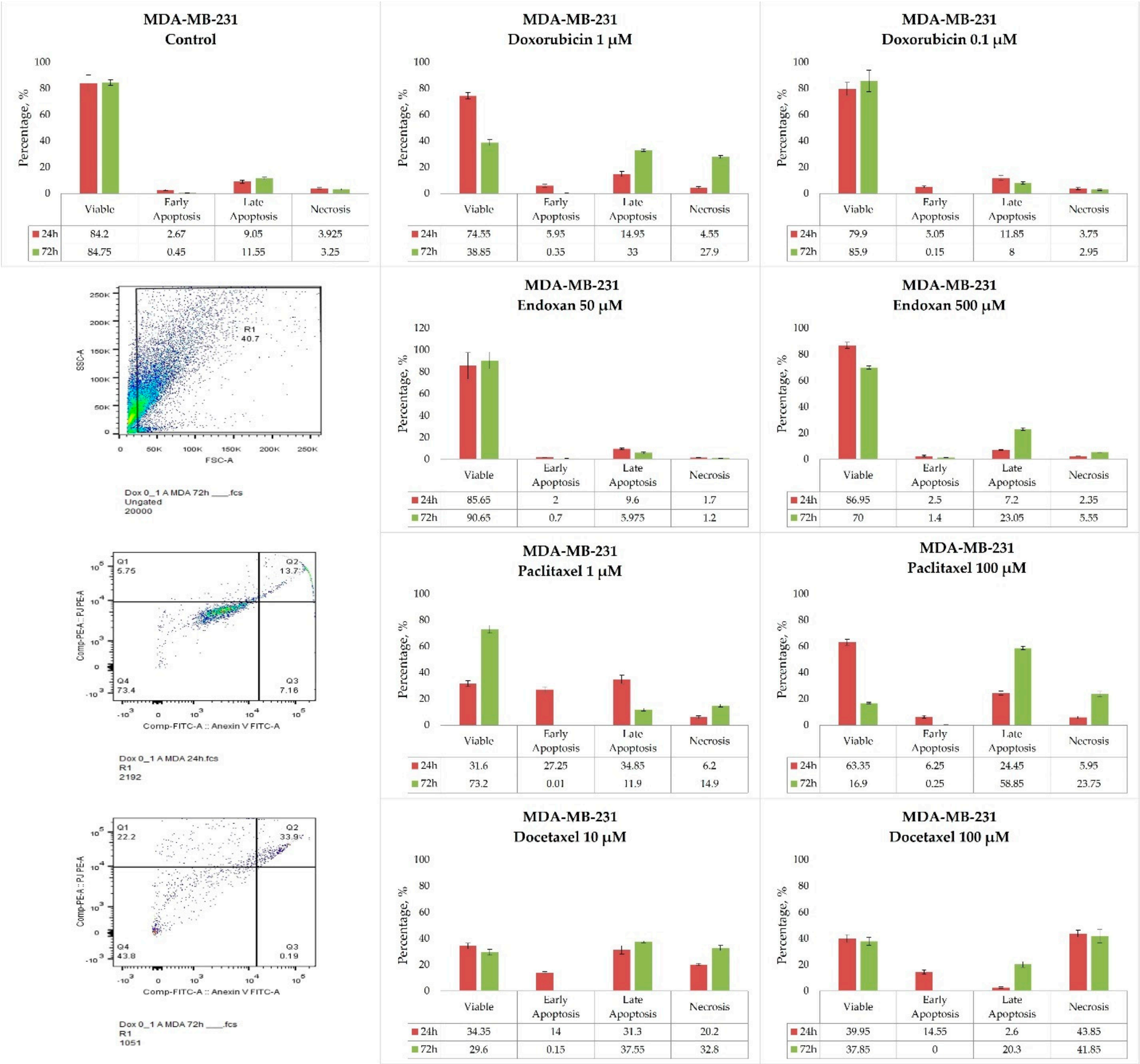

Figure S6. The effects of chemotherapeutics on apoptosis rate in MDA-MB-231 cells. Inlet – Representative dot plots illustrate population of viable (ANNV- PI-), early apoptotic (Ann V+ PI-), late apoptotic (AnnV+ PI+), and necrotic (AnnV- PI+) cells treated with doxorubicin. Apoptosis of untreated as well as chemotherapeutic-treated cells, analyzed by flow cytometry using Annexin V (FITC) and propidium iodide double staining. The data are presented as means ± SEM of a three-independent experiments.

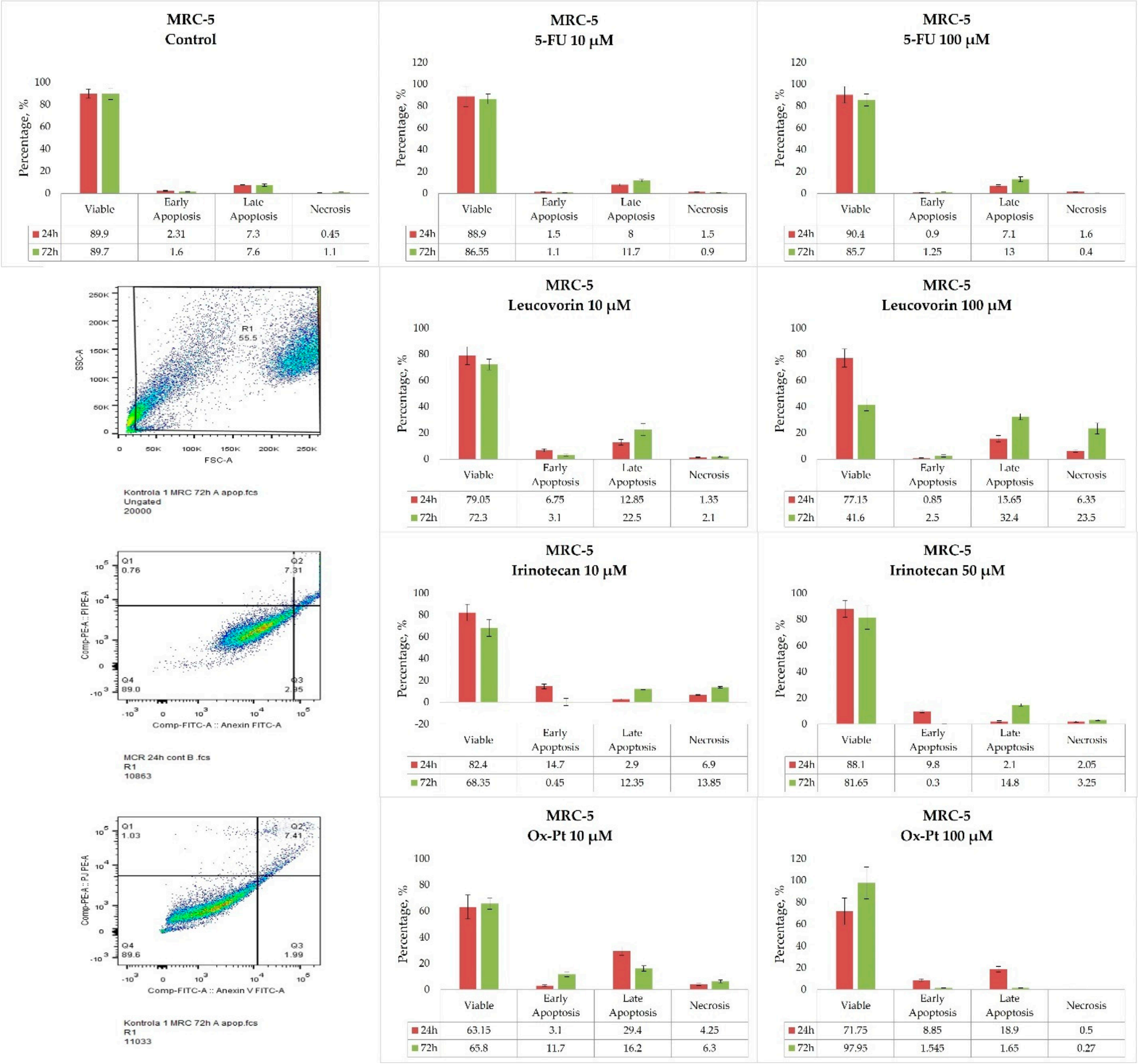

Figure S7. The effects of chemotherapeutics used for colon cancer treatment on apoptosis rate in MRC-5 cells. Inlet – Representative dot plots illustrate population of viable (ANNV- PI-), early apoptotic (Ann V+ PI-), late apoptotic (AnnV+ PI+), and necrotic (AnnV- PI+) control (untreated) cells. Apoptosis of untreated as well as chemotherapeutic-treated cells, analyzed by flow cytometry using Annexin V (FITC) and propidium iodide double staining. The data are presented as means ± SEM of a three-independent experiments.

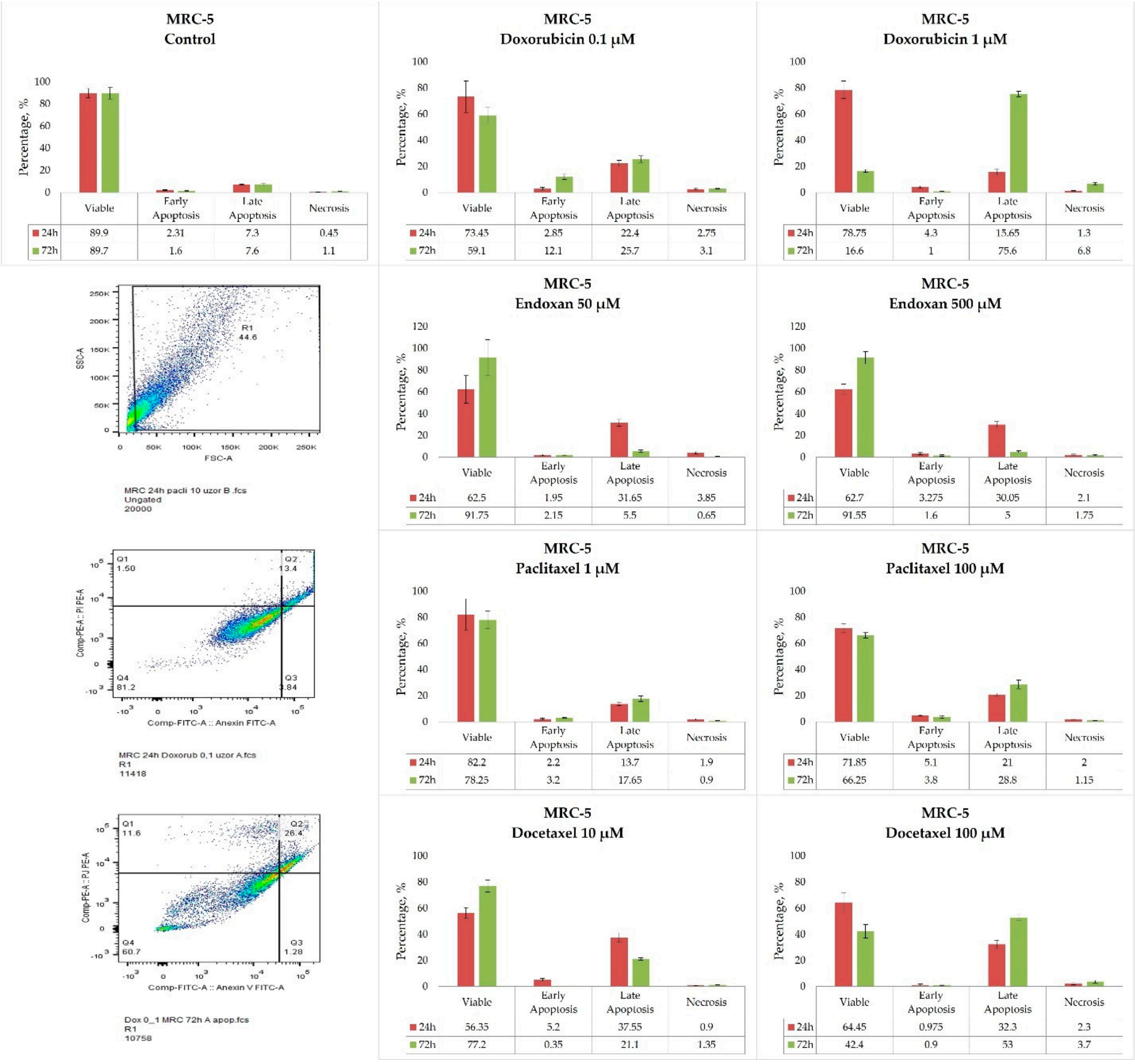

Figure S8. The effects of chemotherapeutics used for breast cancer treatment on apoptosis rate in MRC-5 cells. Inlet – Representative dot plots illustrate population of viable (ANNV- PI-), early apoptotic (Ann V+ PI-), late apoptotic (AnnV+ PI+), and necrotic (AnnV- PI+) cells treated with doxorubicin. Apoptosis of untreated as well as chemotherapeutic-treated cells, analyzed by flow cytometry using Annexin V (FITC) and propidium iodide double staining. The data are presented as means ± SEM of a three-independent experiments.

		HCT-116										MRC-5									
		Bcl-2		*Bax*		*Cas3*		*Cas9*		*Fas*		*Bcl-2*		*Bax*		*Cas3*		*Cas9*		*Fas*	
		**24 h**	**72 h**	**24 h**	**72 h**	**24 h**	**72 h**	**24 h**	**72 h**	**24 h**	**72 h**	**24 h**	**72 h**	**24 h**	**72 h**	**24 h**	**72 h**	**24 h**	**72 h**	**24 h**	**72 h**
**5-FU**	**10 µM**	5.86	4.13	1.10	3.10	1.20	5.50	7.20	0.80	0.70	0.01	4.84	150.64	3.75	1.64	26.21	9.06	1.89	2.46	0.69	0.89
	**100 µM**	5.36	7.26	0.70	1.50	1.40	2.10	17.90	0.50	0.40	0.40	1.66	3.87	36.25	7.38	25.35	7.58	3.22	3.40	1.12	1.14
**Leucovorin**	**10 µM**	5.45	5.98	2.10	0.70	0.46	0.54	0.94	1.24	0.43	0.38	3.94	2.58	0.75	1.68	17.46	40.20	0.66	1.72	0.01	0.03
	**100 µM**	4.80	7.42	5.70	11.40	10.66	3.40	9.72	0.93	1.57	1.96	6.17	17.17	25.21	8.82	64.04	153.92	1.99	8.63	0.02	0.48
**Irinotecan**	**10 µM**	19.90	40.30	0.25	0.92	1.56	13.97	2.19	16.35	0.44	0.16	0.10	4.29	29.67	15.75	3.16	3.48	2.22	0.74	0.02	1.65
	**50 µM**	70.20	142.60	0.23	2.22	2.86	7.62	0.57	8.17	0.58	0.79	20.53	39.72	240.35	14.84	21.61	19.45	14.44	5.72	0.16	2.17
**Ox-Pt**	**10 µM**	309.30	764.30	13.90	2.60	0.01	15.00	35.48	16.81	2.20	3.49	3.16	8.13	1.00	3.14	2.19	39.34	0.58	3.16	0.02	0.01
	**100 µM**	660.30	250.40	29.30	0.60	1.30	5.30	36.23	2.81	2.40	0.85	8.22	24.29	6.80	17.55	2.18	49.38	0.60	0.89	0.05	0.54

		MDA-MB-231										MRC-5									
		Bcl-2		*Bax*		*Cas3*		*Cas9*		*Fas*		*Bcl-2*		*Bax*		*Cas3*		*Cas9*		*Fas*	
		**24 h**	**72 h**	**24 h**	**72 h**	**24 h**	**72 h**	**24 h**	**72 h**	**24 h**	**72 h**	**24 h**	**72 h**	**24 h**	**72 h**	**24 h**	**72 h**	**24 h**	**72 h**	**24 h**	**72 h**
**Doxorubicin**	**0.1 µM**	2.35	22.75	0.05	2.44	0.01	6.27	2.65	9.21	0.01	1.58	17.45	10.77	7.54	10.54	3.29	1.87	3.10	1.83	0.79	1.22
	**1 µM**	0.05	22.88	0.08	1.72	10.72	5.91	3.46	5.05	0.01	1.68	4.91	17.64	6.07	5.30	9.67	11.54	2.83	3.55	0.88	0.77
**Endoxan**	**50 µM**	8.36	1.37	0.70	0.03	0.01	0.03	17.35	0.20	0.01	0.04	8.96	20.75	2.97	5.78	4.23	20.59	2.35	4.71	0.19	1.63
	**500 µM**	1.08	40.00	1.08	27.51	1.02	13.10	3.59	40.00	0.01	0.62	8.13	28.98	7.45	14.91	21.21	29.77	4.66	7.62	0.73	2.21
**Paclitaxel**	**1 µM**	1.72	50.00	0.44	11.20	12.08	45.29	0.77	15.17	0.02	10.16	3.36	12.14	3.36	8.54	1.12	13.04	0.82	2.88	0.02	0.13
	**100 µM**	0.01	67.23	0.56	7.15	4.89	39.12	0.42	5.42	0.01	0.88	6.09	13.61	5.08	9.08	5.94	18.16	1.25	4.36	0.01	0.27
**Docetaxel**	**10 µM**	0.19	3.10	0.37	0.12	4.32	1.61	0.72	0.64	6.99	0.72	5.08	22.50	7.50	9.06	0.03	0.33	1.93	3.14	0.15	0.16
	**100 µM**	8.49	1.34	2.86	1.31	11.38	3.42	1.79	4.25	1.66	0.58	1.40	18.33	7.96	6.23	18.23	6.53	6.87	3.24	0.35	0.25

Figure S9. Relative expression of different genes in HCT-116, MDA-MB-231 and MRC-5 cells after treatment with chemotherapeutics. Results of gene expression are presented as the fold change in mRNA expression in a target sample, normalized to a reference gene and relative to the control sample.

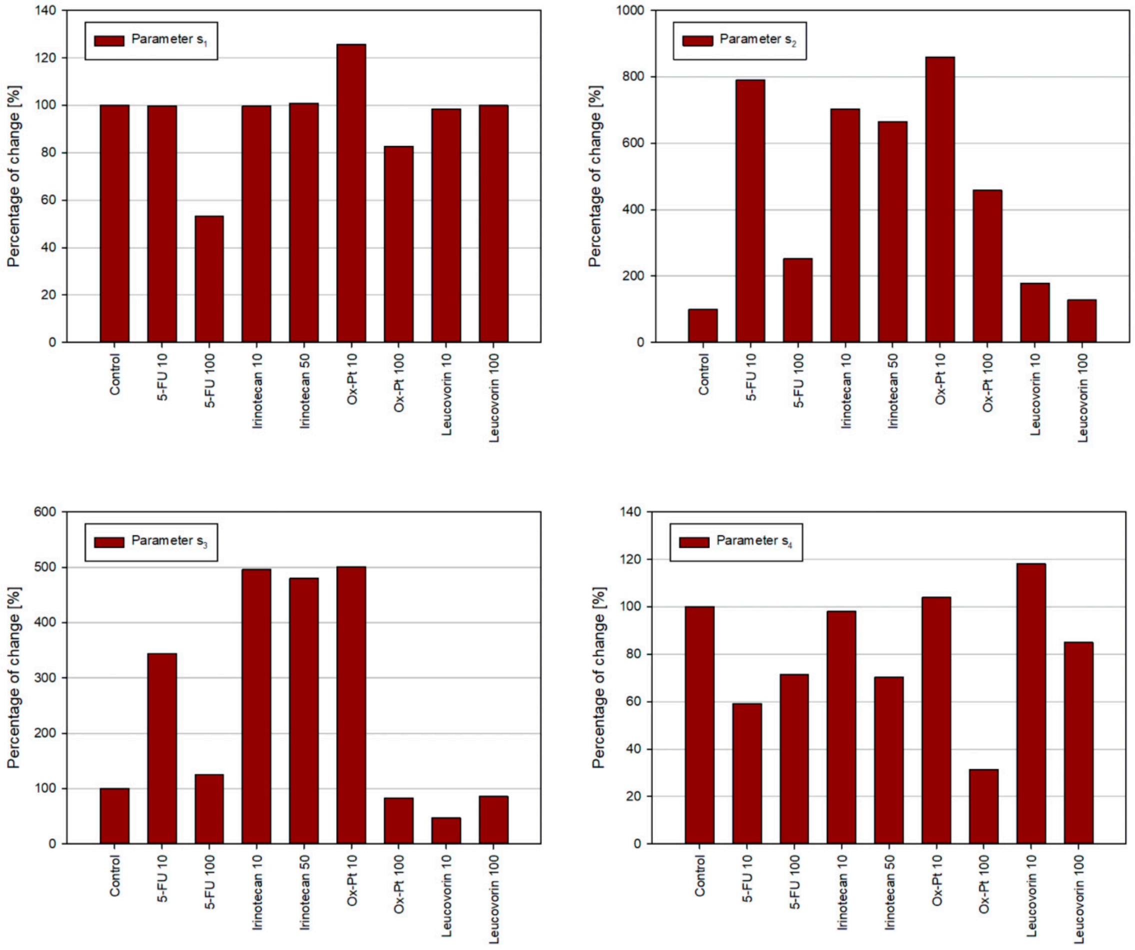

Figure S10. Results of the estimation procedure for the HCT-116 cell line, for all considered treatments, using RTCA experimental data

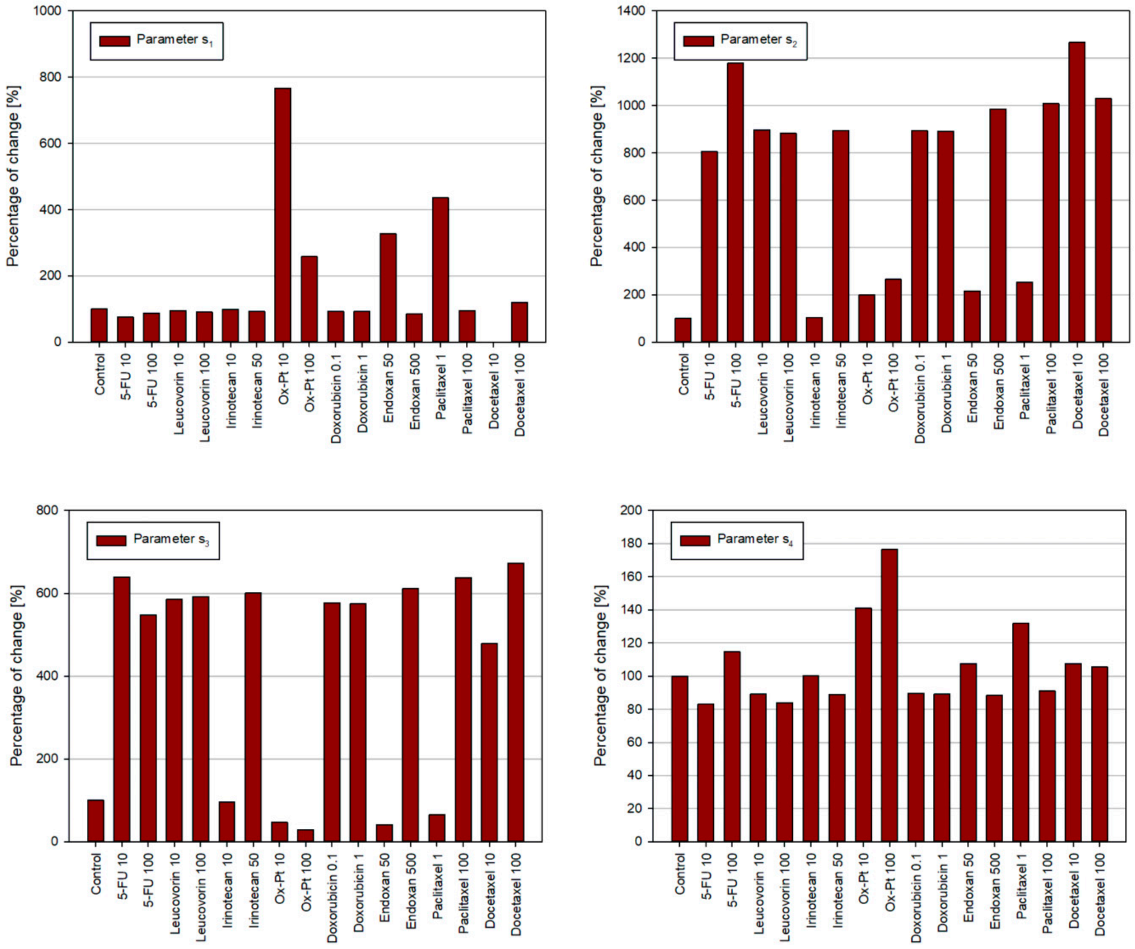

Figure S11. Results of the estimation procedure for the MRC-5 cell line, for all considered treatments, using RTCA experimental data

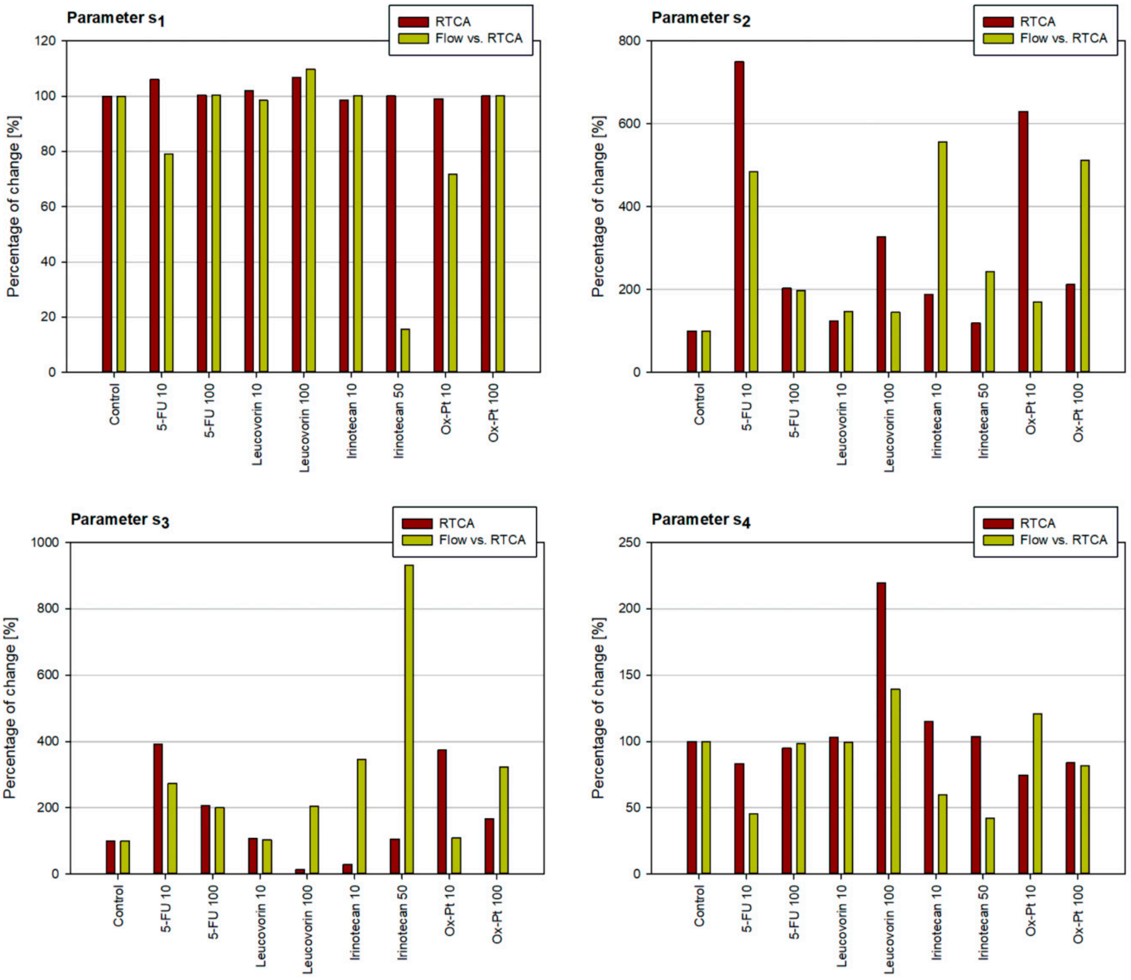

Figure S12. Results of the estimation procedure for the HCT-116 cell line, for all considered treatments, using simultaneously RTCA and Flow experimental data

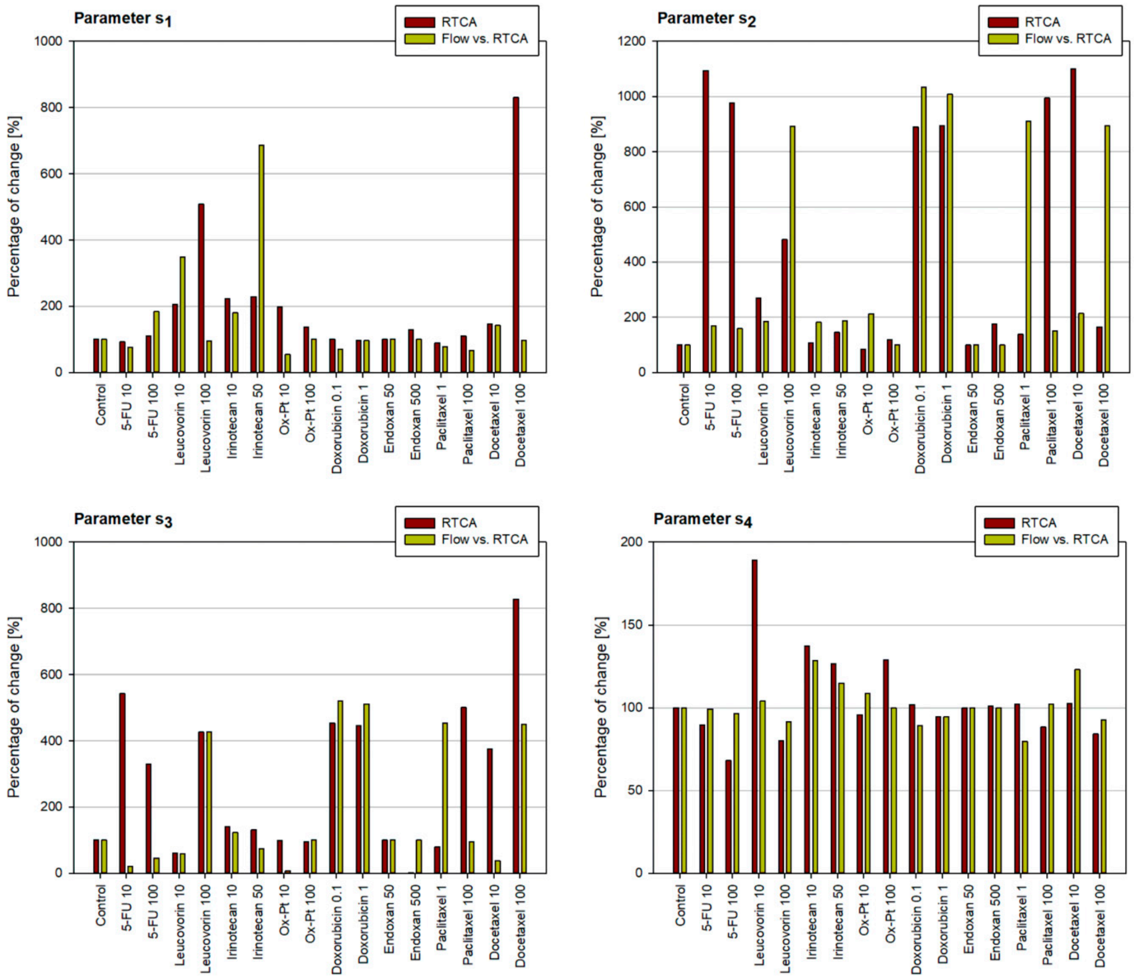

Figure S13. Results of the estimation procedure for the MRC-5 cell line, for all considered treatments, using simultaneously RTCA and Flow experimental data

Table S1. The estimated values of parameters for the HCT-116 cell line for all considered treatments, using RTCA experimental data

Cell line	Parameter s_1	Parameter s_2	Parameter s_3	Parameter s_4
Control	4.58	0.90	0.65	3.21
5-FU 10	4.57	7.12	2.23	1.91
5-FU 100	2.44	2.27	0.81	2.29
Irinotecan 10	4.57	6.34	3.22	3.15
Irinotecan 50	4.62	6.00	3.12	2.25
Ox-Pt 10	5.76	7.74	3.25	3.34
Ox-Pt 100	3.78	4.12	0.54	1.00
Leucovorin 10	4.51	1.61	0.30	3.78
Leucovorin 100	4.58	1.15	0.56	2.73

Table S2. The estimated values of parameters for the MRC-5 cell line for all considered treatments, using RTCA experimental data

Cell line	Parameter s_1	Parameter s_2	Parameter s_3	Parameter s_4
Control	0.58	0.59	0.54	2.32
5-FU 10	0.44	4.74	3.46	1.93
5-FU 100	0.51	6.95	2.96	2.66
Leucovorin 10	0.55	5.28	3.17	2.07
Leucovorin 100	0.53	5.20	3.20	1.94
Irinotecan 10	0.58	0.61	0.52	2.33
Irinotecan 50	0.54	5.27	3.25	2.06
Ox-Pt 10	4.47	1.17	0.25	3.28
Ox-Pt 100	1.50	1.56	0.16	4.09
Doxorubicin 0.1	0.54	5.27	3.12	2.08
Doxorubicin 1	0.54	5.25	3.11	2.07
Endoxan 50	1.91	1.27	0.22	2.49
Endoxan 500	0.49	5.81	3.31	2.05
Paclitaxel 1	2.54	1.49	0.35	3.06
Paclitaxel 100	0.56	5.94	3.45	2.11
Docetaxel 10	0.001	7.46	2.59	2.50
Docetaxel 100	0.70	6.06	3.64	2.45

Table S3. The estimated values of parameters for the HCT-116 cell line for all considered treatments, using the extracted RTCA values and simultaneously the Flow experimental values

Cell line	Parameter s_1		Parameter s_2		Parameter s_3		Parameter s_4	
	RTCA	Flow	RTCA	Flow	RTCA	Flow	RTCA	Flow
Control	4.58		0.90		0.65		3.21	
5-FU 10	4.86	3.62	6.75	4.36	2.54	1.77	2.67	1.46
5-FU 100	4.60	4.60	1.82	1.77	1.34	1.30	3.04	3.16
Leucovorin 10	4.67	4.51	1.11	1.33	0.70	0.67	3.32	3.19
Leucovorin 100	4.89	5.03	2.94	1.31	0.08	1.32	7.05	4.48
Irinotecan 10	4.51	4.58	1.70	5.01	0.19	2.24	3.70	1.92
Irinotecan 100	4.58	0.71	1.07	2.18	0.69	6.05	3.33	1.35
Ox-Pt 10	4.54	3.28	5.67	1.54	2.43	0.71	2.39	3.88
Ox-Pt 100	4.58	4.58	1.91	4.61	1.08	2.09	2.69	2.62

Table S4. The estimated values of parameters for the MRC-5 cell line for all considered treatments, using the extracted RTCA values and simultaneously the Flow experimental values

Cell line	Parameter s_1		Parameter s_2		Parameter s_3		Parameter s_4	
	RTCA	Flow	RTCA	Flow	RTCA	Flow	RTCA	Flow
Control	0.58		0.59		0.54		2.32	
5-FU 10	0.54	0.45	6.45	1.00	2.93	0.11	2.08	2.30
5-FU 100	0.64	1.07	5.76	0.94	1.78	0.25	1.58	2.24
Leucovorin 10	1.20	2.03	1.59	1.08	0.33	0.32	4.39	2.41
Leucovorin 100	2.96	0.56	2.84	5.26	2.30	2.31	1.85	2.13
Irinotecan 10	1.30	1.05	0.62	1.07	0.76	0.66	3.18	2.98
Irinotecan 50	1.33	4.00	0.86	1.11	0.71	0.40	2.94	2.67
Ox-Pt 10	1.15	0.32	0.50	1.25	0.53	0.04	2.22	2.52
Ox-Pt 100	0.80	0.58	0.70	0.59	0.52	0.54	2.99	2.32
Doxorubicin 0.1	0.59	0.41	5.24	6.09	2.45	2.82	2.36	2.07
Doxorubicin 1	0.57	0.56	5.27	5.94	2.41	2.77	2.19	2.19
Endoxan 50	0.58	0.58	0.59	0.59	0.54	0.54	2.32	2.32
Endoxan 500	0.75	0.58	1.03	0.59	0.01	0.54	2.35	2.32
Paclitaxel 1	0.52	0.45	0.81	5.37	0.43	2.45	2.37	1.85
Paclitaxel 100	0.64	0.38	5.86	0.88	2.71	0.52	2.05	2.37
Docetaxel 10	0.86	0.83	6.49	1.26	2.03	0.21	2.38	2.86
Docetaxel 100	4.84	0.56	0.97	5.27	4.48	2.44	1.95	2.15
